# Executive Functions in a Patient with Low-Grade Glioma of the Central Nervous System: A Case Report

**DOI:** 10.3390/tomography10040046

**Published:** 2024-04-18

**Authors:** Manuel José Guerrero Gómez, Ángela Jiménez Urrego, Fernando Gonzáles, Alejandro Botero Carvajal

**Affiliations:** 1 Faculty of Human and Social Sciences, Psychology Program, Universidad de San Buenaventura Cali, Cali 764504, Colombia; manuel.guerrero@u.icesi.edu.co (M.J.G.G.); amjimenezu@usbcali.edu.co (Á.J.U.); 2 Clínica de Occidente, Cali 760001, Colombia; fernando.gonzaleztrujillo@gmail.com; 3 Faculty of Health, Psychology Program, Universidad Santiago de Cali, Cali 760001, Colombia

**Keywords:** Wisconsin card sorting test, executive function, psychology, neuropsychology

## Abstract

Central nervous system tumors produce adverse outcomes in daily life, although low-grade gliomas are rare in adults. In neurological clinics, the state of impairment of executive functions goes unnoticed in the examinations and interviews carried out. For this reason, the objective of this study was to describe the executive function of a 59-year-old adult neurocancer patient. This study is novel in integrating and demonstrating biological effects and outcomes in performance evaluated by a neuropsychological instrument and psychological interviews. For this purpose, pre- and post-evaluations were carried out of neurological and neuropsychological functioning through neuroimaging techniques (iRM, spectroscopy, electroencephalography), hospital medical history, psychological interviews, and the Wisconsin Card Classification Test (WCST). There was evidence of deterioration in executive performance, as evidenced by the increase in perseverative scores, failure to maintain one’s attitude, and an inability to learn in relation to clinical samples. This information coincides with the evolution of neuroimaging over time. Our case shows that the presence of the tumor is associated with alterations in executive functions that are not very evident in clinical interviews or are explicit in neuropsychological evaluations. In this study, we quantified the degree of impairment of executive functions in a patient with low-grade glioma in a middle-income country where research is scarce.

## 1. Introduction

Approximately 30% of primary CNS tumors in children and 6.4% of primary CNS tumors in adults are low-grade gliomas (LGGs), a diverse group of neuroepithelial tumors originating from CNS astrocytes. LGGs are classified as grade 1 or 2 gliomas by the World Health Organization (WHO). While they are benign and grow slowly, many of them rarely improve and frequently develop into higher-grade tumors. Survival times ranging from less than two years to more than ten years are indicative of the diverse clinical effects of LGGs. This diversity is influenced by molecular changes, treatment regimens, tumor features, and patient demographics. Forty percent of patients who are survivors of childhood cancer will experience cognitive compromise between 5 and 10 years after their diagnosis [1,2,3,4,5,6].

A good predictor of cognitive functioning is executive functions because they enable autonomy and adaptation to life in society [7]. This ability to adapt the individual to the environment occurs on the basis of a self-assessment of their own skills, the inhibition of interference, and control over impulsivity when setting goals, planning their achievement, taking initiative, and making thinking more flexible [8].

Executive functions, therefore, are related to social cognition, theory of mind, empathy, interpersonal relationships, recognition of emotions, social perception, social behavior, and social decision making, processes that are vulnerable to brain injury [9,10,11].

Despite the role that executive functions play in the daily lives of patients, a simple search of PubMed up to 7 November 2023 revealed only 196 case reports, none of which evaluated executive functioning in patients. In this sense, we longitudinally evaluated executive function in a patient who presented with a supratentorial tumor based on the grade and type of oligodendroglioma through the treatment process from the preoperative phase to the first phase of complementary treatment with concomitant chemotherapy and radiotherapy.

In this regard, we expected that the implications of oncological diagnosis and treatment would affect cognitive functions such as attention, memory, and executive functioning, since they are the prototypical manifestations of tumors of the central nervous system [12]. Specifically, low-grade gliomas are associated with impaired working memory and are a predictor of executive dysfunction [13]. Cognitive impairment, on the other hand, is common after surgery for patients with gliomas and has a defined severity in relation to exposure to chemotherapy or radiotherapy [7]. Given these implications, we expect to find significant differences in performance on the Wisconsin Card Sorting Test (WCST), the gold-standard test used to assess executive functioning before and after neurological intervention [13]. This case is relevant when assessing the executive functioning of patients from an integrative biopsychosocial perspective across neurology, neuropsychology, and psychology. This perspective tends to be frequently omitted in evaluation and diagnosis given the limitations of the health system. This patient has been followed since 2020, during which critical changes have occurred. The tools used during the preparation of this case report were selected within the framework of neuroimaging techniques (magnetic resonance imaging (iRM) with T1, T2, and FLAIR sequences; spectroscopy and electroencephalography (EEG); and the Wisconsin Card Classification Test (WCST).

### 1.1. Presentation of the Case

A 59-year-old male patient consulted in February 2020 due to a clinical picture with repeated complex partial seizures and subsequent amnesia of the events with complete recovery. As a pathological antecedent, he had chronic hypertension. He was autonomous with activities of daily living, and at the physical examination, he did not experience neurological deficits.

### 1.2. Neurology and Neuroimaging Findings

Structural magnetic resonance imaging (iRM) was performed using T1 gadolinium, T2, and flair sequences. The results obtained are described below according to the capture date of each iRM (from oldest to most recent).

**Figure 1:**
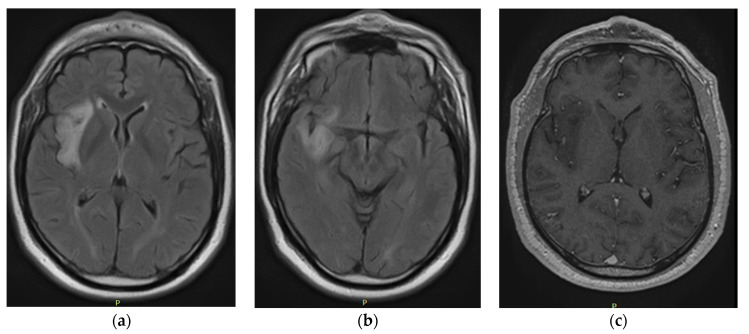
February 2020: iRM FLAIR axial (**a**,**b**) and T1 gadolinium (**c**).

The initial paraclinical findings (Figure 1), such as magnetic resonance imaging of the brain that was performed in February 2020, were abnormal. An image with hyperintensity of the signal was found; it was oval, well defined, and hypocaptant, and it was located in the right periinsular territory with extension to the right temporal lobe. The lesion had a diameter of 4.4 × 1.5 cm and slight perilesional edema, with a compressive effect on the lenticular nucleus, as well as white matter with nonspecific signal hyperintensities associated with gliosis foci. No abnormal uptake of contrast agent was observed. The probable diagnosis was a nodular lesion in the perisylvian territory and right temporal area compatible with low-grade glioma.

An extension study with magnetic resonance spectroscopy performed with multivoxel and univoxel spectroscopy sequences revealed, in the area of interest, an evident decrease in the peak density of N acetylaspartate with an increase in the peak density of choline and creatine, as well as a Cho/Cr index of 2.94 and a Cho/Cr of 0.52, which are associated with probable low-grade astrocytoma-type gliomas. The following complementary studies were carried out to rule out other pathologies: a normal lumbar puncture (February 2020) with a gram without microorganisms, a glucose concentration of 62.5 mg/dL, a protein concentration of 29.2 mg, a pH of 8, a negative India ink, and a nonreactive serology vdrl; the measured tumor markers returned normal results. These included testosterone (593), alpha-fetoprotein (1.85), carcinoembryonic antigen (1.87), ca. 19–9 (27.45), and psa (2.02). An electroencephalogram (EEG) was recorded (February 2020), and the results were normal.

The treatment plan was open surgery with the objective of wide resection and obtaining tissue for pathological study. As an anticonvulsant treatment, he received 1000 mg of levetiracetam daily and 1000 mg of valproic acid valcote ER daily. The patient and his relatives did not accept the surgical procedure, the patient was discharged, and the observation process continued in the outpatient clinic for 18 months without variations in health conditions or controlled epilepsy.

**Figure 2:**
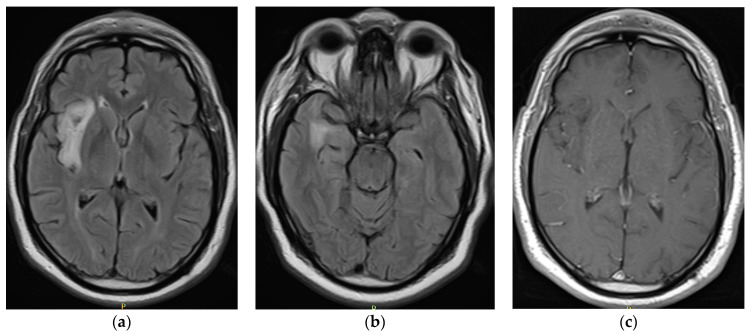
September 2021: iRM FLAIR axial (**a**,**b**) and T1 gadolinium (**c**) images.

In the September 2021 control (Figure 2), brain resonance revealed changes in the volume of the tumor, and cortical and subcortical involvement was detected in the right insular region due to an area of intensity alteration that was hypointense in T1 and hyperintense in T2 and FLAIR, without gadolinium enhancement and predominantly peripheral diffusion restriction; this region measured 33.5 × 50 × 21 mm, which is compatible with low-grade glioma. The patient and his family decided to authorize the surgery, which was performed in October 2021. Surgical intervention was carried out through partial resection of the lesion, with no subsequent neurological deficits or postoperative complications.

**Figure 3:**
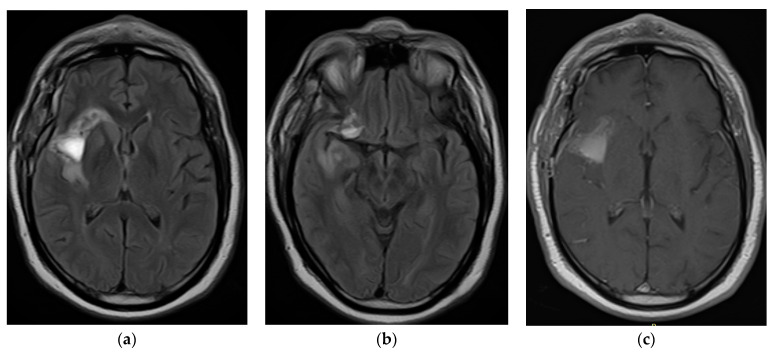
November 2021: Post-surgery axial flair iRM (**a**,**b**), T1 gadolinium (**c**).

The control MRI in November 2021 (Figure 3) described postoperative changes in the right insular region with the presence of hematoma and the persistence of residual tumor lesions prior to the surgical intervention area. Pathology revealed a glial neoplasm with characteristics concordant with oligodendroglioma: olig2+, idh 1+, ki67 less than 3%, p53-, without necrosis, without significant mitotic activity, and without endothelial proliferation. In consensus, the treating physicians considered that the patient had risk factors such as age group, persistence of the tumor lesion due to the last MRI performed, and an increase in the volume of the lesion during the period of approximately 18 months under observation; thus, the pathology revealed a low-grade glial neoplasm. The patient had to be treated for a high-grade neoplasm with a Stupp protocol: chemotherapy with temozolomide and concomitant radiotherapy and subsequent adjuvant therapy with temozolomide. The patient was stable with periodic controls and received the treatment protocol at the institution on an outpatient basis.

## 2. Findings from the Psychological Clinical Interview

A clinical interview was carried out with the patient’s caregiver, a person with whom he shared a home and who has remained with him most of the time during the subject’s life cycle. The procedure consisted of two clinical interview sessions, which made it possible to collect systematic information about the patient’s functioning in vital areas and related to the family of origin and presence, religious beliefs, work and financial stressors, relationships, and social relationships. Subsequently, relevant information was provided when discussing alterations in social cognition. The presence of medical, pharmacological, psychiatric, psychological, family, and personal antecedents was explored.

The clinical psychological interview process did not reveal a history of neurological, oncological, or psychopathological pathologies in the family. The caregiver reported a history of the couple’s therapy process due to the pre-existence of relational difficulties between both individuals prior to the appearance of the patient’s clinical condition. He came from a family with parents who died at an early age and whose loss at age ten had a significant impact in terms of the lack of instrumental and emotional support from their parental figures. His wife described the patient’s father as authoritarian, given the small amount of affection he had experienced during childhood.

The patient’s literacy could reach the third grade, and the patient had adequate reading and writing skills and remarkable skills for calculating according to what his wife said. This indicates deterioration in the visual health of the patient after surgery and deterioration in calculation skills. He had worked since childhood in agriculture and mining, and he developed his early life in precarious economic conditions due to the loss of his parental figures. He was characterized by having a wide social circle in the course of his life, being a charismatic and receptive individual. These conditions changed as the glioma treatment progressed.

The symptomatology of the tumor was evidenced by nocturnal seizures in 2018, which lasted approximately ten seconds per episode and increased in frequency over time with daytime and nighttime episodes. The diagnosis was formalized in 2019, and surgery to resect the tumor began in November 2021, accompanied by oral chemotherapy and radiation therapy in January 2022. The caregiver reported noticeable changes in behavior since the appearance of the tumor. Increased isolation and disconnection from the environment, constant episodes of sadness, emotional dullness, poor communication, and reserved temperament were observed. The inhibition of attention toward environmental stimuli, for example, toward conversations in social gatherings, was notorious. These observations made it possible to note possible deterioration in aspects of the patient’s behavior and social perception toward others, in addition to apathetic behavior and emotional alterations.

Defects in attention, short-term memory, and working memory were evident from the beginning of the treatment, according to the impressions narrated by the caregiver. Prior to the appearance of the tumor, he highlighted the outstanding performance of the patient’s memory in general terms, as well as a remarkable ability to perform calculations. Some signs of anterograde amnesia were observed when he began to develop difficulties in recalling episodic memories about conversations and people. In terms of orientation, the patient showed a tendency toward spatial disorientation, although orientation to people and in time was still preserved.

## 3. Neuropsychological Findings

The approach to obtaining the neuropsychological profile of the patient was carried out by means of the Wisconsin Card Classification Test (WCST), a useful tool to assess cognitive strategies in the face of changes in the environment—that is, cognitive flexibility. The NRS-2002 is a sensitive test for assessing executive function [13]. This consists of four stimulus cards, with four differentiated geometric figures: circles, crosses, triangles and stars. These cards have three qualities: color, shape, and number. The procedure begins by providing the subject with a set of 128 cards (or 64 cards for the brief application), which must be arranged one by one, below one of the four cards initially provided through the conceptualization of a criterion (shape, number or color). The criterion and the correct decision are established based on the feedback provided by the evaluator.

The frontal neuropsychological damage questionnaire developed by Flores, Ostrosky, and Lozano (2014) and provided by the second edition of the Neuropsychological Battery of Executive Functions and Frontal Lobes (BANFE-II) was used. The SFRS is a behavioral scale applied to caregivers with 40 items and is composed of a Likert-type scale ranging from 0 to 5 (1: almost never; 2: rarely; 3: sometimes; 4: frequently; 5: almost always).

Regarding executive functioning, the results are presented as failures for the maintenance of attitude, learning to learn and perseverance. Cognitive flexibility is expressed in learning to learn and perseverance. However, working memory fails to maintain this attitude. The findings are shown by means of pre- and post-surgical sequences of tumor resection (Table 1).

Prior to tumor resection, the borderline was located at a typical score of 79. This indicates that the percentage of patients in the normative group exceeds 8% (8%). Three out of six categories were completed on the WCST, with thirty-five (35) attempts to complete the first, which resulted in a performance in learning to learn, which reflects a performance that exceeded 16% of the normative population. Despite the percentile in which the individual is located, the number of attempts necessary to complete the first category and the number of categories completed allow us to glimpse a cognitive flexibility to improve compared to the normative group. The categories completed were located between the sixth and tenth percentiles, exceeding between six and ten percent of the population, together with a percentile location of between two and five percent with respect to the normative population, around the number of attempts to complete the first category.

The deterioration in cognitive flexibility seven days after tumor resection was notable. On the post-surgical WCST, a moderate deterioration was observed, and the performance of the participant translated to a typical score of 68 on the perseveration test. This implies a considerable deficit with respect to the performance of the pre-surgical test. The deficit in cognitive flexibility is notable in the retest, since he did not manage to complete any category corresponding to the WCST. This places him in a percentile that does not exceed the performance of one percent of the normative population (<1%). The patient required 128 items to complete the first category of the test without success, a situation that places him between the second and fifth percentiles in contrast to the normative group (2–5%).

There was no possibility of calculating learning to learn due to the performance of the subject, so the absence of calculations in this domain and the increase in perseverations indicate that the cognitive flexibility of the patient was impaired. Therefore, it was impossible for him to consider and structure alternative strategies to solve the problem of the task. The role of cognitive flexibility would suggest possible deficits in working memory, given the difficulties in keeping information in place and practice consciousness and operate on it, actively modifying itself according to its need. The legitimacy of the findings in terms of WCST performance is specified according to testimony from the patient’s caregiver about the cognitive and longitudinal changes associated with the appearance and treatment of the glioma. In this sense, the frontal lobe inventory seeks to objectify the behaviors of the patient by inquiring with the caregiver about it and receives 13 points out of a possible 40, which places him close to the minimum global functional commitment. Therefore, we consider the possibility unlikely that there was a falsified result in the WCST, given the contrasting sources of information mentioned on the existence of cognitive impairment. These findings are related to neuroimaging, the caregiver’s testimony, and the frontal damage questionnaire.

**Figure 4:**
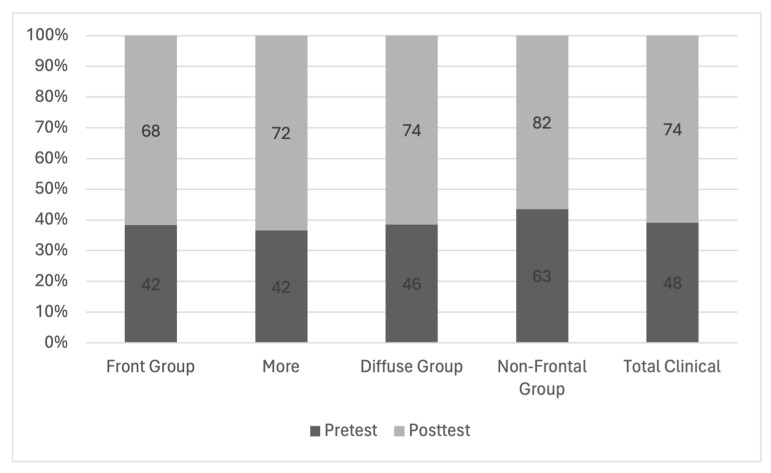
The similarity of patient performance with clinical groups considered by the WCST depicting cognitive deterioration by percentage.

The results regarding perseverations allowed us to consider possible cognitive deficits in the patient during the pre-test, which were more evident during the post-test. The performance during the first test was 47.5% closer to the performance reported by the WCST clinical group. The increase in the deficit became more evident in the post-test, in which the closeness increased to 73.7%, similar to that of the total clinical group (Figure 4).

The increase in the percentage of similarity between the pre-test and the post-test in groups of patients with brain injuries that made up the clinical group is highlighted. Since we compared the patient group with the frontal group, after the recession of the tumor and after chemo- and radiological therapy, an increase in the similarity of the patient to each of the clinical comparison groups was observed: frontal, diffuse, frontal or clinical.

## 4. Discussion

Executive functioning, evidenced by perseverance, learning to learn, and failure to maintain one’s attitude, was more impaired than what can be observed in neurological or psychological interviews. The use of complementary neuropsychological assessment instruments allows us to account for the state of impairment of executive functions due to the action of the tumor, treatment, or surgery. Starting from this context, the objective of this report is to describe the executive functioning of a 59-year-old adult neurocancer patient through a pre–post design.

Executive functioning is crucial for patients to observe and control their behavior and adapt it to the contextual demands that derive from the evolution of diagnosis, treatment, family, work, and personal changes, which together are key factors for adherence to treatment [7]. In turn, assessments of cognitive status in neuro-oncological patients should consider other risk factors that contribute to the deterioration observed, such as age, the extent of surgery, the use of antiepileptic drugs, tumor variants associated with different reported survival outcomes (benign astrocytoma-type or glioblastoma-like malignant forms), and early tumor progression [8].

Tumors are generated in areas where both local damage and disorganization of cognitive networks occur, and the most affected domains in patients are attention, memory, and executive functions [3,4]. Direct cancer treatment applied early in life to the CNS compromises brain development by reducing the volume of the cerebral cortex and altering connectivity; additionally, biological changes can lead to brain vascular damage, a reduction in the population of stem cells, oxidative stress, and inflammation. Ionizing radiation causes cellular senescence, epigenetic changes, and alterations in DNA repair. Chemotherapy drugs such as methotrexate generate epigenetic changes and inhibit the reduction of free radicals. Cyclophosphamide induces ovarian failure during early menopause, and a reduction in estrogen affects the protection of these cells in the cardiovascular system in patients [1].

Studies that compare temozolomide-type chemotherapy with radiotherapy do not reveal improvements in aspects such as quality of life, cognitive improvement, or progression-free survival. Studies on cognitive impairment include radiation therapy; proton therapy schemes reduce the dose of brain tissue exposed to radiation compared to photon therapy, which seeks to reduce structural damage and preserve cognitive abilities. Studies that compare both therapies and the application of neurocognitive tests do not find differences in the time of commitment initiation [8,9,10].

Radiation therapy currently avoids irradiating structures such as the hippocampus, and in patients with metastases, the use of stereotaxic radiosurgery has increased as a treatment option to reduce the risk of deterioration. According to an expert consensus review, no well-developed studies have supported the use of a drug that significantly improves cognition in the neuro-oncological population [10].

Therapeutic interventions focused on the preservation and improvement of cognitive ability involve both cognitive rehabilitation therapy and drug treatment [3]. At present, vorasidenib is a promising drug for improving survival and reducing the time needed for subsequent intervention in patients with low-grade gliomas, which allows patients and families to find effective intervention options for individuals with observed cognitive and executive decline [11].

Neuropsychological tests applied to neuro-oncological patients have been used to assess executive functions, memory, attention, and processing speed [12,13]. There are different tests to apply in patients, for example, the European Organization for the Research and Treatment of Brain Cancer, which applies the revised Hopkins Verbal Learning Test (HVLT), which measures memory domains and the evocation of achieved learning; the TMT (Trail Making Test), which assesses attention, speed, and mental flexibility; the Controlled Oral Words Association (COWA) test, which assesses the spontaneous production of words in restricted search conditions; others, which are mini-mental tests; and the Montreal Cognitive Assessment (MoCA) [13].

From this perspective, the Wisconsin Card Classification Test (WCST), one of the tools most commonly used at the investigative level, is useful because of its degree of sensitivity in discriminating executive functions between clinical and nonclinical samples; therefore, low functioning suggests frontal lobe pathologies. This test provides an explanatory framework for why subjects with frontal pathologies, as well as in areas peripheral to the frontal lobe, obtain unfavorable results in the test. What is observed in behavior is therefore a decrease in the functioning of social cognition and interpersonal relationships: ToM, recognition of emotions, empathy, social decision making, social perception, and social behavior [10].

This phenomenon allows us to provide an explanatory framework for the behavioral changes evidenced by the caregiver of the patient throughout the process and through the notorious degradation of cognitive abilities in the patient, as is the case for calculation and memory. Parallel events to the apathetic behavioral tendency may be associated with diminished social perception, emotional dullness, and sadness, accompanied by isolation and disconnection from what happens in their social environment as part of the impoverishment of social behavior.

The foregoing discussion is pertinent insofar as the postoperative MR findings indicated residual tumor lesions anterior to the resection area and changes in the right insula with the presence of hematoma. Notorious particularity occurs when considering the insular region as an essential interoceptive substrate for emotional sensitivity and processing and social cognition, while it is valuable for the attribution of emotional and social salience to the situation [9,10,11].

## 5. Conclusions

Using the Wisconsin Card Classification Test (WCST), the performance of executive functioning during neurological or psychological interviews is difficult to quantify with respect to neurotypical functioning. We emphasize that visualizing EFs allows patients greater metacognitive and emotional knowledge about changes related to diagnosis and treatment, which are elements that help clinicians understand adherence to treatment.

Simultaneously, we highlight the value of this report because it allowed us to explore in detail the characteristics of the patient’s specific executive functioning, which contributed to the integration of information at a qualitative and quantitative level, which is useful for making inferences about the characterization of the subject’s executive performance and developing a descriptive and explanatory panorama of the current cognitive profile of the subject.

Finally, although the main purpose of the present study was to describe the executive functioning of this patient, we found that social cognition is affected by brain damage [10,11]. However, these processes are limited because phenomena such as empathy, ToM, or social perception are not commonly recognized in Colombia, which has few such scales of the population. This is one of the essential aspects to be improved upon in future studies and is essential for neuropsychological evaluation in the future.

## Figures and Tables

**Table 1:** Perseverations, learning to learn, and failure to maintain one’s attitude.

Test	RC	RP	ND	Performance	CC (PC)	FMA	AA
Pre	78	79	Intermediate Deterioration	Borderline	6–10%	>16	N/P
Post	48	68	Moderate deterioration	Poor	<1%	>16	N/P

RC: correct responses, PR: perseverative responses (typical score), ND: level of impairment; CC: complete categories; FMA: failures.

## Data Availability

All the data are included in the paper.
